# Validation of Quantitative Flow Ratio-Derived Virtual Angioplasty with Post-Angioplasty Fractional Flow Reserve—The QIMERA-I Study

**DOI:** 10.3390/jcdd11010014

**Published:** 2023-12-31

**Authors:** Ignacio J. Amat-Santos, Giorgio Marengo, Juan Pablo Sánchez-Luna, Carlos Cortés Villar, Fernando Rivero Crespo, Víctor Alfonso Jiménez Díaz, José María de la Torre Hernández, Armando Pérez de Prado, Manel Sabaté, Ramón López-Palop, José Miguel Vegas Valle, Javier Suárez de Lezo, Clara Fernandez Cordon, Jose Carlos Gonzalez, Mario García-Gómez, Alfredo Redondo, Manuel Carrasco Moraleja, J. Alberto San Román

**Affiliations:** 1 Cardiology Department, Hospital Clínico Universitario de Valladolid, 47003 Valladolid, Spain; 2 Cardiology Department, Hospital Miguel Servet, 50009 Zaragoza, Spain; 3 Cardiology Department, Hospital La Princesa, 28006 Madrid, Spain; 4 Cardiology Department, Hospital Álvaro Cunqueiro, 36312 Vigo, Spain; 5 Cardiology Department, Hospital Marqués de Valdecilla, 39008 Santander, Spain; 6 Cardiology Department, Hospital Clínico Universitario, 24071 León, Spain; 7 Cardiology Department, Hospital Clinic Universitari, 08001 Barcelona, Spain; 8 Cardiology Department, Hospital Virgen de la Arrixaca, 30120 Murcia, Spain; 9 Cardiology Department, Hospital de Cabueñes, 33203 Gijón, Spain; josemivv@secardiologia.es; 10 Cardiology Department, Hospital Reina Sofía, 14004 Córdoba, Spain; 11 Cardiology Department, Hospital Universitario de Santiago, 15706 Santiago de Compostela, Spain

**Keywords:** coronary physiology, fractional flow reserve, quantitative flow ratio, virtual angioplasty

## Abstract

**Background:** Quantitative flow ratio (QFR) virtual angioplasty with pre-PCI residual QFR showed better results compared with an angiographic approach to assess post-PCI functional results. However, correlation with pre-PCI residual QFR and post-PCI fractional flow reserve (FFR) is lacking. **Methods:** A multicenter prospective study including consecutive patients with angiographically 50–90% coronary lesions and positive QFR results. All patients were evaluated with QFR, hyperemic and non-hyperemic pressure ratios (NHPR) before and after the index PCI. Pre-PCI residual QFR (virtual angioplasty) was calculated and compared with post-PCI fractional flow reserve (FFR), QFR and NHPR. **Results:** A total of 84 patients with 92 treated coronary lesions were included, with a mean age of 65.5 ± 10.9 years and 59% of single vessel lesions being the left anterior descending artery in 69%. The mean vessel diameter was 2.82 ± 0.41 mm. Procedural success was achieved in all cases, with a mean number of implanted stents of 1.17 ± 0.46. The baseline QFR value was 0.69 ± 0.12 and baseline FFR and NHPR were 0.73 ± 0.08 and 0.82 ± 0.11, respectively. Mean post-PCI FFR increased to 0.87 ± 0.05 whereas residual QFR had been estimated as 0.95 ± 0.05, showing poor correlation with post-PCI FFR (0.163; 95% CI:0.078–0.386) and low diagnostic accuracy (30.9%, 95% CI:20–43%). **Conclusions:** In this analysis, the results of QFR-based virtual angioplasty did not seem to accurately correlate with post-PCI FFR.

## 1. Introduction

In the last few decades, physiology-guided coronary revascularization has changed the treatment approach of coronary artery disease (CAD), carrying better prognostic evaluation and improved outcomes compared to angiography-based revascularization [1,2,3,4,5]. To achieve an extended use of physiology without FFR (Fractional Flow Reserve) adenosine-hyperemia-related issues, several non-hyperemic pressure ratios (NHPR) as instantaneous Wave-free Ratio (iFR), diastolic resting pressure ratio (dPR) or resting full-cycle ratio (RFR) have been developed [6,7,8,9]. Nevertheless, despite guidelines recommendations, the use of physiology in daily practice remains below 30%, mostly due to increased procedural duration, inappropriate reimbursement policies, and guidewire-associated risks [10,11,12].

To overcome these issues, computational techniques have been developed and nowadays the most validated one is Quantitative Flow Ratio (QFR) (QAngio XA 3D QFR, Medis Medical Imaging System; Leiden, the Netherlands) [13]. QFR is a computational technique that combines three-dimensional quantitative coronary angiography (3D-QCA) with computational fluid dynamics to calculate the functional significance of coronary stenosis. This full angiographic-based method allows a rapid and precise fractional flow reserve (FFR) estimation without the need for coronary wiring and/or hyperemia induction.

Excellent correlation between QFR and FFR values have been reported, even better than between FFR and non-hyperemic indexes [13,14,15,16,17,18] and this also applies to post-PCI functional evaluation. In this context, like with FFR, a QFR value > 0.90 has been related to a better prognosis [19,20,21], but with QFR the post-PCI functional evaluation can be estimated beforehand just by virtually simulating the correct resolution of the coronary stenosis. This is called “virtual angioplasty” and might be crucial to plan the best strategy for revascularization and to potentially predict procedural outcomes.

Such simulated reconstruction can be easily performed with QFR software, but its use has not been validated yet. Therefore, we sought to determine the correlation between QFR-obtained “virtual angioplasty” and post-PCI hyperemic and non-hyperemic indexes.

## 2. Methods

### 2.1. Design and Study Population

**Figure 1:**
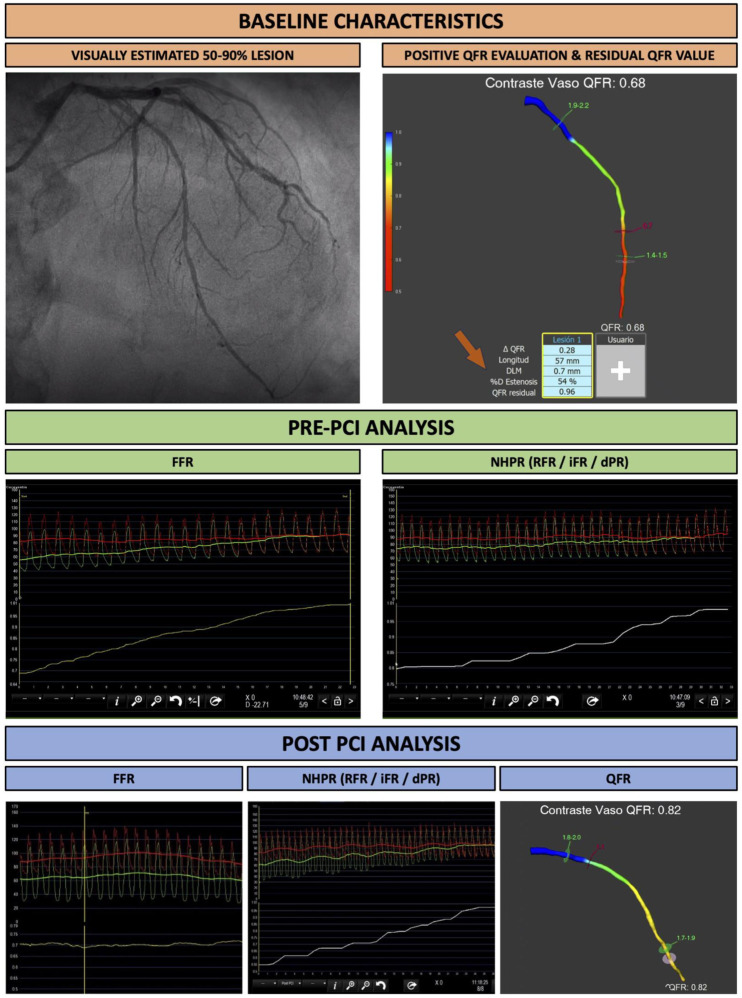
Study flowchart.

This is a multicenter and prospective study including consecutive patients undergoing coronary angiography and physiological assessment with QFR, dPR, RFR, iFR and FFR. In order to be enrolled, patients had to undergo coronary angiography for stable angina, unstable angina, NSTEMI or LVEF deterioration. The angiographic characteristics of included patients were the following: all subjects had a visually estimated coronary stenosis between 50 and 90% in a vessel with a size > 2 mm in the stenosed segment. All patients underwent baseline QFR evaluation and only subjects with a baseline positive QFR (defined as QFR ≤ 0.80) were included. Patients with ostial left main lesions, intrastent restenosis and severe aortic stenosis were excluded from the study, along with patients with severe asthma or severe chronic obstructive pulmonary disease, allergy to contrast media or adenosine, ST-elevation myocardial infarction, negative QFR evaluation (defined as QFR > 0.8) or when calculating QFR was not suggested because of low angiographic quality or poor contrast filling. All patients underwent physiological assessment with QFR and FFR before and after PCI, along with one NHPR (iFR, dPR or RF) (Figure 1). For wire-based assessment, FFR was considered the gold standard in case of disagreement. Two independent operators performed the QFR-based virtual angioplasty simulation. These operators were unaware of both the wire-based physiological analysis result and the PCI strategy. At the end of the procedure, QFR-based virtual angioplasty results were compared with post-PCI wire-based physiological values. This analysis was conducted in compliance with the Declaration of Helsinki and after approval by the local ethics committees.

### 2.2. Study Endpoint

The main endpoint of this analysis was to determine the accuracy of QFR virtual angioplasty (with residual QFR values) and its correlation with post-PCI hyperemic and non-hyperemic indexes.

### 2.3. Quantitative Flow Ratio Analysis

Angiographic acquisitions followed the recommendations provided in the FAVOR II study [17] to allow proper QFR calculation: for each lesion two projections (with a fluoroscopy rate of 15 frames per second) were acquired to minimize the risk of shortening and overlying structures, allowing a complete vessel visualization. Intracoronary nitrates were administrated before angiographic acquisitions. QFR analyses were performed by two certified operators blinded to wire-based physiological analysis results and the PCI strategy. During the pre-PCI QFR evaluation of the index coronary was performed, along with the residual QFR (representing the same vessel QFR after PCI of the target lesion), which was automatically calculated by the 1.3 version of QFR software. Only vessels with a positive QFR were included in this study and QFR evaluation of the target vessel was repeated after PCI.

### 2.4. Hyperemic and Non-Hyperemic Indexes Analysis

Wire-based invasive physiological measurements were performed using PressureWire (Abbott Vascular, Santa Clara, CA, USA), Optowire II (Opsens medical, Quebec, Canada) or OmniWire (Philips, Amsterdam, The Netherlands), according to the tool availability in each center. After guiding catheter (6F–7F) engagement, the pressure wire was advanced, equalized to aortic pressure and then positioned at distal segments of the diseased artery. Intracoronary nitrates were administrated before measurements.

First of all, NPHR (iFR or dPR or RFR) was obtained after distal placement of the wire and repeated during wire pullback. Finally, the wire was advanced again and FFR was calculated after hyperemia induction with adenosine—either by intracoronary bolus (200–300 μg) or intravenous perfusion (140 μg/kg/min). After pullback and after final FFR measurements, the wire was retired up to the catheter tip and the absence of drift was checked. If pressure drift was detected all the measurements were repeated. The position of the wire was recorded for all measurements and used as a distal reference for QFR calculation. All these analyses were performed before and after PCI.

### 2.5. Statistical Analysis

Categorical variables were presented as absolute frequencies and percentages. Continuous variables were presented as mean ± standard deviation. Data were analyzed per patient for baseline characteristics and vessels for functional and angiographic evaluation. Correlation between residual QFR and FFR, and NHPR was obtained by Pearson’s correlation analysis. Sensitivity, specificity and accuracy were calculated to evaluate the capacity to predict post-PCI good functional results (FFR > 0.90 or NHPR > 0.95) with pre-PCI residual QFR (cut-off value 0.9). All tests were two sided. Differences were statistically significant when the *p*-value was < 0.05. Statistical analysis was performed using R software, version 3.6.1 (R Project for Statistical Computing).

## 3. Results

### 3.1. Baseline Characteristics of Study Population

A total of 84 patients with 92 coronary lesions were included. Baseline characteristics are summarized in Table 1. Mean age was 65.5 ± 10.9 years, with 17% of women. Hypertension was present in 83% of patients and dyslipidemia in 89%; 65% of patients had a previous MI and 65% underwent a prior PCI. In 39% of cases, coronary angiography was performed for non-culprit lesion evaluation in non-ST-elevation acute myocardial infarction (NSTEMI), whereas 38% of patients were studied for stable angina, 13% for unstable angina and 10% for LVEF deterioration. A Canadian Cardiovascular Society angina grade of 1 or 2 was found in 92% of patients.

### 3.2. Procedural Characteristics

The main procedural characteristics are summarized in Table 2. A radial approach was used in 92% of cases, the mean fluoroscopy time was 17 min [12–26 min] and the mean contrast administration was 239 mL [178–280 mL]. In 59% of the patients, there was single-vessel disease, while two- and three-vessel disease was found in 33% and 5%, respectively. The left anterior descending artery was the most common target vessel (69% of patients), followed by the Circumflex (16%) and the right coronary artery (16%). The median angiographic severity of the target lesion was 60% [52–70%], with 59% of bifurcation lesions and 9% of severe calcifications. The mean vessel diameter in the treated segments was 2.82 ± 0.41 mm.

Pre-dilation was performed in 58% of cases and post-dilatation in 38%, with 78% of procedures requiring single-stent implantation, and an overall mean number of stents implanted of 1.17 with a mean stent diameter and length of 2.9 ± 0.45 mm and 27 mm [19–35 mm], respectively. Procedural success (defined as a post-PCI TIMI 3 flow in the target vessel without loss of side branches with ≥2 mm diameter) was achieved in all the procedures.

### 3.3. Physiological Analysis

The main measurements obtained before and after PCI through quantitative coronary analysis are reported in Table 3. All patients underwent QFR and FFR analysis. A NHPR was performed for 77 coronary lesions (83.7%), with iFR used in 25 (32.5%), DPR in 16 (20.8%) and RFR in 36 (46.8%). In the remaining 15 patients (16.3%), NHPR was not performed due to the operator’s choice or a specific cath lab’s local routine practice.

According to inclusion criteria, all lesions had a positive QFR result, with a mean baseline QFR value of 0.69 ± 0.12. Mean baseline FFR and NHPR values were, respectively, 0.73 ± 0.08 and 0.82 ± 0.11. The estimated residual QFR (“virtual angioplasty”) was automatically calculated as 0.95 ± 0.05.

### 3.4. Correlation between Virtual Angioplasty and Post-PCI FFR, QFR and NHPR

**Figure 2:**
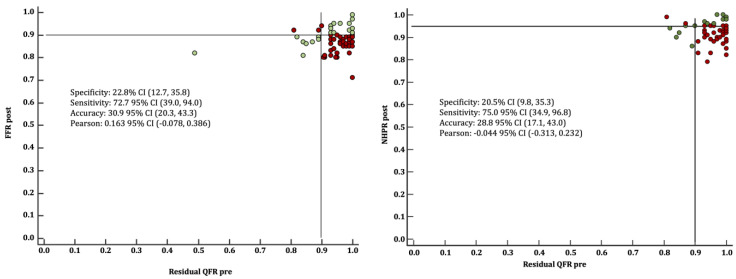
Correlation between virtual angioplasty and post-PCI FFR. **Left panel.** Scatter Plot showing correlation between virtual angioplasty (pre-PCI residual QFR) and post-PCI FFR values (green: good correlation; red: poor correlation). **Right panel.** Scatter Plot showing correlation between virtual angioplasty (pre-PCI residual QFR) and post-PCI NHPR values (green: good correlation; red: poor correlation).

The correlation between virtual angioplasty by QFR and post-PCI FFR, QFR and NHPR are shown in Table 4. The mean FFR pre-PCI was 0.73 ± 0.08 and increased to 0.87 ± 0.05 after PCI. The correlation between residual QFR and post-PCI FFR values was 0.163 (95% CI: 0.078, 0.386). The mean NHPR values pre-PCI were 0.82 ± 0.11 and increased to 0.92 ± 0.05 after PCI. The correlation between residual QFR and post-PCI NHPR values was −0.044 (95% CI: −0.313, 0.232). The mean QFR pre-PCI was 0.69 ± 0.12 and increased to 0.95 ± 0.05 after PCI. The correlation between residual QFR and post-PCI QFR values was 0.058 (95% CI −0.175, 0.284). As reflected in Figure 2, virtual angioplasty presented a diagnostic accuracy of 30.9% (95% CI: 20.3–43.3%) when considering 0.9 as a threshold for both pre-PCI residual QFR and post-PCI FFR. In addition, virtual angioplasty presented a diagnostic accuracy of 28.8% (95% CI:17.1–43%) when considering 0.95 as a threshold for post-PCI NHPR and a 0.9 threshold for pre-PCI residual-QFR.

## 4. Discussion

In this multicenter registry, we aimed to investigate the efficacy and accuracy of virtual PCI as assessed by QFR in clinical practice. Additionally, we provide a correlation analysis between pre-PCI estimated residual QFR or “virtual angioplasty” and post-PCI values assessed by common hyperemic and non-hyperemic indexes. The main finding of our analysis can be summarized as follows: (i) pre-PCI residual QFR values showed low accuracy and poor correlation when compared with post-PCI FFR and NHPR; and (ii) the prognostic relevance of residual QFR as estimated at baseline could not be excluded despite the lack of adequate correlation with pressure wire analysis. The presence of calcified plaques precluding from correct expansion might be behind these findings. Indeed, residual QFR might represent the potential optimal result of PCI suggesting that FFR and NHPR values could be optimized using plaque modification devices or better stent sizing. Also, as shown in Figure 1, the presence of diffuse disease might condition the final stent length differing from what was estimated by virtual angioplasty.

Since the publication of the DEFER trial in 2001 [5], it is well known that coronary lesions should not be treated with PCI when there is a negative value at functional evaluation given that medical therapy alone is related to fewer adverse cardiovascular events compared to PCI at an extended 15 years of follow up [22]. In addition, strong evidence is questioning the role of PCI in chronic coronary syndrome even with functionally positive coronary lesions [3], because it may not be related to clear mortality benefit [23]. This is radically changing the interventional cardiologist’s approach when facing an angiographically ambiguous coronary lesion and is supporting a widespread diffusion of coronary physiology tools. Also, in the context of post-PCI evaluation, a physiology-based strategy showed better results when compared with visual estimation [24], and post-PCI values of FFR ≥ 0.90 and iFR ≥ 0.95 have been related to improved outcomes [19,25]. Likewise, as reported by a recent meta-analysis, a post-PCI FFR below 0.90 is encountered in nearly 60% of angioplasties and was associated with a higher risk of cardiac death and target vessel myocardial infarction [26].

Despite this robust evidence to date, in a substantial proportion of cases, this game-changing approach is still not provided to the patients, causing the unnecessary invasive treatment of a large amount of harmless coronary stenoses and, in the case of coronary angioplasty, a potential suboptimal result.

The main limitations to the wider use of intracoronary physiology are larger procedural times, wire issues (mostly related to the low crossability and trackability of the pressure wire) and problems due to adenosine administration.

To overcome these concerns, QFR represents a solid alternative, not being burdened by the need for a coronary wire or by hyperemia induction [14]. In comparison with standard wire-based intracoronary physiology, QFR has been related to similar performances and showed better results compared with standard anatomical assessment [14,15,18]. Even in the post-PCI setting QFR seems an effective technique and can be used to drive prognostic intervention decision making, with lower post-PCI QFR values accurately predicting adverse clinical outcomes [27]. As discussed by Chen et al., post-PCI QFR assessment showed a solid predictive value over future adverse cardiovascular events and this finding was confirmed by another large meta-analysis, with a 58% risk reduction per each 0.1 increase in the post-PCI QFR value [28,29]. In this context, compared with wire-based physiology, QFR has a theoretical additional advantage, as it may allow to predict beforehand the functional result of the angioplasty. This could be crucial in the daily catheterization laboratory practice to detect the subgroup of lesions that will not significantly improve after PCI, avoiding overtreatment and the use of unnecessary stents (with all the procedure-related complications and the long-term risk of intra-stent restenosis) [30]. Recently, the AQVA trial demonstrated that QFR-based virtual PCI was superior to conventional angiography-based PCI at achieving an optimal post-PCI physiology result (defined as a post-PCI QFR value ≥ 0.90). QFR-based virtual PCI strategy changed the operators’ procedural plan in 25% of the cases and was not associated with either longer procedures or a higher amount of contrast or radiation dose [31].

Unfortunately, despite the clear prognostic benefit, this beforehand approach has not yet been validated as a routine approach for PCI decision making and this could be also due to a lack of evidence comparing residual QFR with post-PCI FFR, which still represents the gold standard for post-PCI functional evaluation.

To date, very little evidence is available about the association between residual QFR and post-PCI FFR. This topic was initially assessed by Rubimvura et al. in a sub-analysis of the DOCTORS (Does Optical Coherence Tomography Optimize Results of Stenting) study and, in this case, the authors showed a good correlation between these 2 indexes (r = 0.68) [32]. However, severe calcifications and diffuse vessel disease were exclusion criteria and this could have conditioned the results, leading to a general overestimation of the correlation shown. Indeed, residual QFR predicts the results of a technically successful angioplasty and does not fully consider potential issues (such as stent under-expansion in severely calcified vessels) that can worsen the PCI results and impair actual post-PCI functional values. Another retrospective analysis with wider inclusion criteria was provided by Van Diemen et al. [33], with all kinds of lesions suitable for post-PCI FFR and residual QFR evaluation. Correlation between residual QFR and FFR was good (r = 0.56), but again these results did not fully reflect common clinical practice, because post-PCI functional evaluation was performed only in the case of “successful angioplasty” defined by the operators.

In this context, our study tried to investigate the actual correlation between pre-PCI residual QFR and wire-based post-PCI functional evaluation (including NHPR) in all-comers. In contrast with the previous data, residual QFR showed low accuracy and poor correlation with post-PCI FFR and NHPR values and this might be due to the more extensive inclusion criteria. As discussed, residual QFR aims to predict functional post-PCI values in case of ideal angioplasty and can be very accurate in case of successful not complicated PCI. Conversely, the correlation between this virtual index and post-PCI FFR is worsened by the inclusion of vessels more likely to undergo a complex PCI (such as calcified vessels or diffusely diseased), whose final results may be harder to predict beforehand. Moreover, even in case of good final PCI result, post-PCI values could be impaired by not-clinically significant distal plaque embolization and by minimal vessel dissection, which can slow coronary flow compared with pre-PCI baseline QFR measurements, and this may hamper the accuracy of virtual PCI by residual QFR, especially in the acute setting of ACS. Finally, as discussed by Lee et al., QFR values can be hindered in case of coronary tandem lesions or diffuse disease because of complex geometry and lesion crosstalk phenomenon, which can significantly affect QFR accuracy in detecting hyperemic flow velocity between each stenosis [34].

The main limitation of this study is due to the lack of case selection and intracoronary imaging use that might help to understand the lack of correlation between QFR and FFR. Also, the limited sample size will require further validation of the main findings.

In conclusion, although QFR-based virtual angioplasty can help to tailor the treatment strategy according to single vessel pre- and post-PCI characteristics, simplifying the procedure, and improving long-term outcomes, this tool does not seem to have adequate accuracy and correlation compared with post-PCI FFR assessment, which remains the actual gold standard. Further studies with larger sample sizes and optimized software are needed to continue exploring this approach.

## Figures and Tables

**Table 1:** Baseline characteristics of the study population.

Baseline Characteristics		Global Study Population (N = 84)
**Age—years**		65.52 ± 10.87
**Gender (female)—no. (%)**		14 (17)
**Hypertension—no. (%)**		59 (83) *
**Dyslipidemia—no. (%)**		55 (89) *
**Diabetes mellitus—no. (%)**		28 (78) *
**Smoking—no. (%)**		42 (50)
**Chronic kidney disease—no. (%)**		11 (29) *
	Creatinine (mg/dL)	0.89 (0.76–1.12)
	Dyalisis—no. (%)	1 (4) *
**Prior MI—no. (%)**		19 (50) *
**LVEF (%)**		57 (46–60)
**Peak Troponin (ui/mL)**		805 (135–4000)
**Prior PCI—no. (%)**		31 (65) *
**Peripheral artery disease—no. (%)**		4 (14) *
**Clinical presentation—no. (%)**		
	Stable angina	32 (38)
	Unstable angina	11 (13)
	Non-ST-elevated MI	33 (39)
	LVEF deterioration	8 (10)
**Medications**		
	Aspirin	81 (96) *
	Ticagrelor	28 (33) *
	Clopidogrel	47 (56) *
	Prasugrel	7 (8) *
	Direct oral anticoag.	7 (8) *
	Warfarine	1 (1) *
**Killip Kimball**		
	I–II	46 (92) *
	III–IV	4 (8) *^
**CCS class**		
	I	9 (36) *
	II	14 (56) *
	III	2 (8) *

Values are expressed as means ± standard deviation (SD), medians (interquartile ranges), or n (cumulative %, * valid %). ^ among the Killip III–IV patients, all patients had a Killip III class.

**Table 2:** Procedural characteristics.

Baseline Characteristics		Global Study Population (N = 84)
**Vascular access**		
	Radial	78 (92)
	Femoral	5 (5)
	Cubital	1 (1)
**Procedural duration (min)**		69 (52–86)
**Contrast dose**		239.5 (178–280)
**Fluoroscopy dose**		98 (55–1383)
**Fluoroscopy time**		17.43 (12–26)
**Target lesion** ^		
	Left anterior descending	48 (69) *
	Circumflex	11 (16) *
	Right coronary artery	11 (16) *
	Left main	3 (4) *
**Diameter reference**		2.82 ± 0.41
**Lesion characteristics**		
**Stenosis diameter (%)**		60 (52–70)
**Length (mm)**		20 (14–31)
**Number of lesions**		
	1	45 (59) *
	2	25 (33) *
	3	4 (5) *
**Type of lesion** °		
	Calcified	8 (9)
	Sequential	7 (8)
	Bifurcation	55 (59)
**NHPR indexes used**		
	RFR	36 (46.8)
	DPR	16 (20.8)
	iFR	25 (32.5)
**Number of implanted stents**		1.17 ± 0.46
	0	1 (1)
	1	72 (78)
	2	12 (13)
	3	2 (2)
**Stent diameter (mm)**		2.93 ± 0.45
**Stent length (mm)**		27 (19–35)

Values are expressed as means ± standard deviation (SD), medians (interquartile ranges) or n (cumulative %, * valid %). ^ data about target lesion location were available for 73 lesions out of 92. ° data about lesion type were available for 70 lesions out of 92.

**Table 3:** Pre- and post-PCI results of physiological and angiographic analyses.

	Baseline	Post-PCI
**QFR**	0.69 ± 0.12	0.95 ± 0.05
**Residual QFR**	0.95 ± 0.07	/
**Hyperaemic index**		
**FFR**	0.73 ± 0.08	0.87 ± 0.05
**Non-hyperaemic indexes**		
**NHPR**	0.82 ± 0.11	0.92 ± 0.05

**Table 4:** Correlation between virtual angioplasty and post-PCI indexes.

		Lin’s CCC	Pearson ρ	*p*-Value
**Residual QFR**	**QFR post**	0.050 (−0.150, 0.246)	0.058 (−0.175, 0.284)	0.627
	**FFR post**	0.090 (−0.044, 0.221)	0.163 (−0.078, 0.386)	0.185
	**NHPR post**	−0.035 (−0.255, 0.187)	−0.044 (−0.313, 0.232)	0.757

## Data Availability

Data are available under request.

## Abbreviations

CAD	Coronary artery disease
dPR	Diastolic Resting Pressure Ratio
FFR	Fractional Flow Reserve
iFR	Instantaneous Wave-free Ratio
NPHR	Non-hyperemic pressure ratio
NSTEMI	Non-ST-elevation myocardial infarction
OMT	Optimal Medical Therapy
PCI	Percutaneous coronary intervention
QFR	Quantitative Flow Ratio
RFR	Resting Full-cycle Ratio
STEMI	ST-elevation myocardial infarction
